# Supplementary material for: Redox-Cycling “Mitocans” as Effective New Developments in Anticancer Therapy
Source: Int J Mol Sci. 2023 May 8;24(9):8435. doi: 10.3390/ijms24098435 (PMC10179378; doi:10.3390/ijms24098435)
Supplement: Supplementary file 1 [file ijms-24-08435-s001.zip › ijms-2306739-supplementary.pdf]

Supporting information

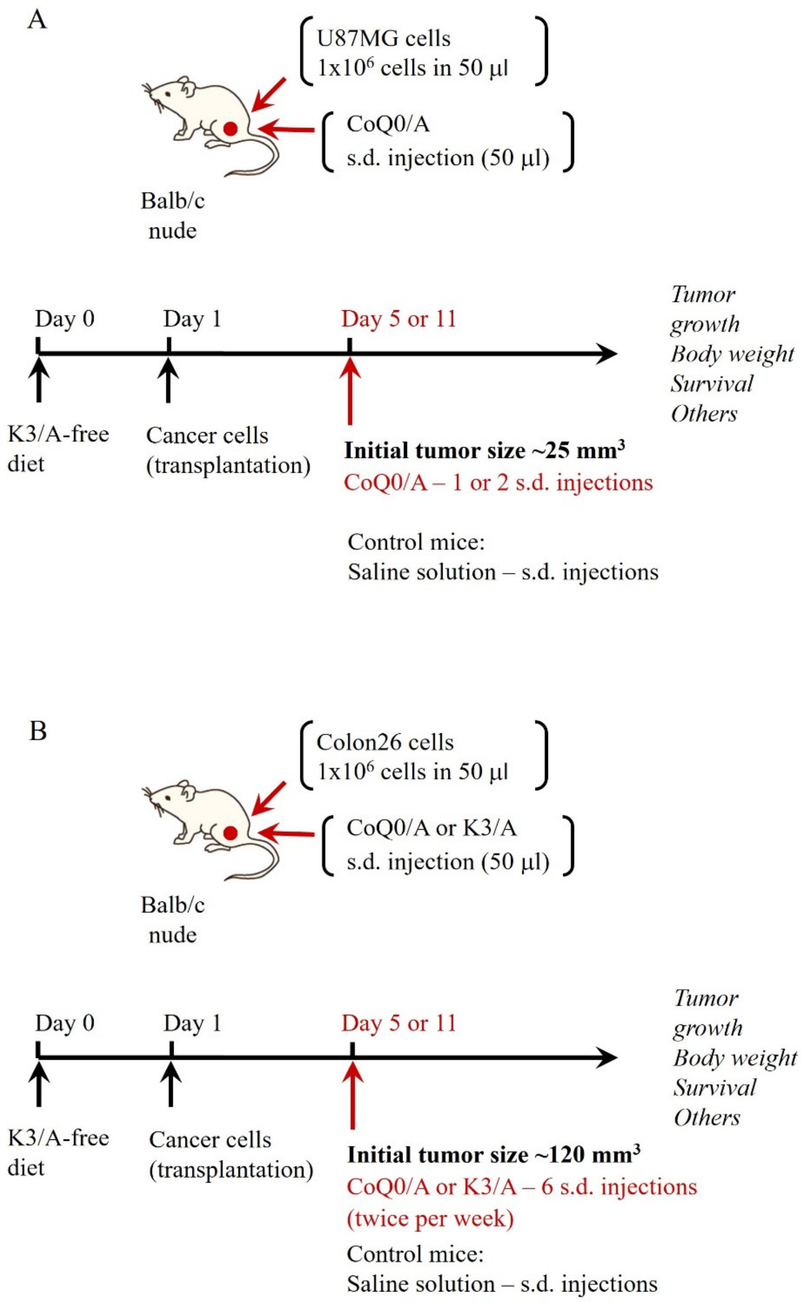

Figure S1. Experimental design in vivo: glioblastoma (A) and colon cancer (B) hind paw xenografts and subdermal (s.d.) injection of drug near the tumor.

Table S1. Effects of ascorbate, quinone, and their combination on cell viability and proliferation, expressed as percentage of control (untreated) cells – synergism between both substances.

Concentrations (μM/μM)	1/100	2/200	3/300	5/500	10/1000	20/2000
Ascorbate only	100	100	100	100	83	68
ThymoQ<br>ThymoQ/A<br>P-value	n.d.	98.5<br>76.4<br>P<0.05<br>Synergistic	83.8<br>57.3<br>P<0.01<br>Synergistic	70.0<br>42.4<br>P<0.01<br>Synergistic	37.5<br>29.6<br>P<0.05	34.3<br>30.0<br>n.s.
DMBQ<br>DMBQ/A<br>P-value	102.0<br>82.4<br>P<0.05<br>Synergistic	94.6<br>67.5<br>P<0.01<br>Synergistic	67.5<br>43.1<br>P<0.01<br>Synergistic	47.3<br>35.5<br>P<0.05<br>Synergistic	35.0<br>30.0<br>n.s.	30.2<br>29.3<br>n.s.
MMBQ<br>MMBQ/A<br>P-value	n.d.	103.5<br>78.2<br>P<0.05<br>Synergistic	91.5<br>59.2<br>P<0.01<br>Synergistic	86.9<br>42.9<br>P<0.001<br>Synergistic	53.8<br>36.4<br>P<0.01<br>Additive	34.3<br>30.0<br>n.s.
CoQ0<br>CoQ0/A<br>P-value	100.8<br>72.6<br>P<0.01<br>Synergistic	74.0<br>53.1<br>P<0.05<br>Synergistic	51.4<br>41.6<br>P<0.05<br>Synergistic	37.0<br>32.4<br>n.s.	32.6<br>24.4<br>n.s.	27.3<br>23.5<br>n.s.
CoQ1<br>CoQ1/A<br>P-value	n.d.	n.d.	100.5<br>88.5<br>n.s.	85.0<br>80.5<br>n.s.	80.4<br>75.3<br>n.s.	74.5<br>58.0<br>P<0.05
CoQ10<br>CoQ10/A<br>P-value	n.d.	n.d.	100.7<br>100.0<br>n.s.	102.4<br>102.8<br>n.s.	102.5<br>98.5<br>n.s.	95.0<br>86.8<br>n.s.
K3<br>K3/A<br>P-value	n.d.	n.d.	102.0<br>77.6<br>P<0.05<br>Synergistic	96.7<br>60.7<br>P<0.01<br>Synergistic	82.0<br>33.2<br>P<0.001<br>Synergistic	49.2<br>33.3<br>P<0.05<br>Additive
K1<br>K1/A<br>P-value	n.d.	n.d.	101.3<br>100.9<br>n.s.	101.0<br>96.0<br>n.s.	85.0<br>78.2<br>n.s.	72.4<br>58.0<br>P<0.05<br>Additive
K2<br>K2/A<br>P-value	n.d.	n.d.	98.5<br>100.2<br>n.s.	95.0<br>92.3<br>n.s.	85.5<br>81.4<br>n.s.	78.5<br>80.0<br>n.s.
AtovaQ<br>AtovaQ/A<br>P-value	n.d.	n.d.	94.0<br>103.3<br>n.s.	80.3<br>92.1<br>P<0.05	60.5<br>67.5<br>n.s.<br>Antagonistic	40.3<br>45.0<br>n.s.<br>Antagonistic
BNQ<br>BNQ/A<br>P-value	103.2<br>92.1<br>n.s.	82.6<br>63.5<br>P<0.05<br>Synergistic	56.2<br>41.8<br>P<0.05<br>Synergistic	48.3<br>34.1<br>P<0.05<br>Synergistic	38.8<br>35.9<br>n.s.	35.4<br>35.5<br>n.s.

Average data and P values corresponding to Figure 2D are included in the Table.
Synergistic effect: When the effect of Q/A is higher than the sum of the effects of Q and

A administered alone.
Additive effect: When the effect of Q/A is equal to the sum of the effects of Q and A administered alone.
Antagonistic effect: When the effect of Q/A is lower than the sum of the effects of Q and A administered alone.
